# Access to colorectal cancer screening for Pakistani immigrants in Norway – a qualitative study

**DOI:** 10.1186/s12913-024-11275-7

**Published:** 2024-07-11

**Authors:** Nadia Iqbal, Paula Berstad, Marit Solbjør, Esperanza Diaz, Elżbieta Czapka, Solveig Hofvind, Sameer Bhargava

**Affiliations:** 1 Cancer Registry of Norway, Norwegian Institute of Public Health, P.O. Box 5313, Majorstuen, Oslo, 0304 Norway; 2 Institute of Health and Society, Faculty of Medicine, University of Oslo, Oslo, Norway; 3 Department of Public Health and Nursing, Faculty of Medicine and Health Science, NTNU - Norwegian University of Science and Technology, P.O. Box 8905, Trondheim, N-7491 Norway; 4 Department of Global Public Health, University of Bergen, P.O. Box 7804, Bergen, NO-5020 Norway; 5 Department of Health and Function, Western Norway University of Applied Sciences, Bergen, Norway; 6 Department of Social Sciences, University of Gdańsk, Bażyńskiego 8, Gdańsk, 80-309 Poland; 7 Department of Health and Care Sciences, The Arctic University of Norway, P.O. Box 5313, Tromsø, 0304 Norway; 8 Department of Oncology, Akershus University Hospital, Lørenskog, Norway

**Keywords:** Colorectal cancer, Screening participation, Immigrants, Health inequalities

## Abstract

**Background:**

The Norwegian colorectal cancer (CRC) screening program started in May 2022. Inequalities in CRC screening participation are a challenge, and we expect that certain groups, such as immigrants, are at risk of non-participation. Prior to the start of the national screening program, a pilot study showed lower participation rates in CRC screening among immigrants from Pakistan. These immigrants are a populous group with a long history in Norway and yet have a relatively low participation rate also in other cancer screening programs. The purpose of this study was to identify and explore perspectives and factors influencing CRC screening participation among immigrants from Pakistan in Norway.

**Materials and methods:**

In this study we used a qualitative study design and conducted 12 individual interviews with Pakistani immigrants aged between 50 and 65 years. The participants varied in terms of gender, age, education, work, residence time in Norway and familiarity with the Norwegian language and culture. We performed thematic analysis with health literacy as a theoretical framework to understand Pakistani immigrants’ perspectives on CRC screening.

**Results:**

We identified four main themes: Health-related knowledge, the health care system, screening, and social factors. Within these themes we identified several factors that affect Pakistani immigrants’ accessibility to CRC screening. These factors included knowledge of the causes and development of cancer, sources of health-related information, the general practitioner’s role, understanding of screening and the intention behind it, language skills and religious beliefs.

**Conclusion:**

There are many factors influencing Pakistani immigrants’ decision of participation in CRC screening. The roles of the general practitioner and adult children are particularly important. Key elements to improve accessibility to CRC screening and enable informed participation for Pakistani immigrants are measures that improve personal and organizational health literacy.

**Supplementary Information:**

The online version contains supplementary material available at 10.1186/s12913-024-11275-7.

## Introduction

Colorectal cancer (CRC) is the third most common type of cancer globally, accounting for about 10% of all cancers [1]. In Norway, CRC is the fourth most common type of cancer and the second most common cause of cancer death [2]. Since CRC symptoms often are unspecific and usually present at a late stage of the disease, clinical manifestations often indicate advanced stage disease beyond the prospect of cure. The 5-year relative survival rate after surgery exceeds 95% for early stage CRC (stage I) while it is slightly above 30% for stage IV [3].

In order to detect CRC in an early stage and reduce the specific mortality from the disease, organized screening programs for CRC are recommended by the European Union [4]. A nationwide screening program, ColorectalScreen Norway, was launched in Norway in May 2022. The target group is individuals aged 55 to 65, and the screening method is fecal immunochemical tests (FIT) every second year for a 10-year period. FIT consists of a stool sample taken at home and shipped to a laboratory in a prepaid envelope. A positive FIT prompts a colonoscopy which aims to detect and remove precursor lesions and early-stage cancer. According to European guidelines, a minimum acceptable participation rate for FIT screening is 45%, while a desired participation is at least 65% [5].

In many countries, cancer screening uptake is lower in socio-economically deprived groups and in immigrants [6, 7]. Studies from Norway have shown a substantially lower participation rate among immigrants compared to non-immigrants in mammographic and cervical cancer screening, and in the pilot for CRC screening [8–10]. Prior to startup of ColorectalScreen Norway, a pilot was conducted in two counties in the period 2012–2023 [11]. The participation rate was 28% among immigrants born in Pakistan in the first round of FIT screening in the pilot project, as compared to 60% among non-immigrants [10]. Despite the intention for equal access [12], participation disparities suggest otherwise. Several barriers and reasons for non-participation are suggested, including lack of awareness of CRC and CRC screening, lack of recommendations from physicians, psychological factors (for instance fear, anxiety and shame), cultural/religious factors and sociodemographic factors (including language barrier, income and gender) [13].

A heterogenous group of immigrants from all countries and continents comprise 16% of Norway’s total population of 5.4 million people [14]. There are more than 22,000 immigrants from Pakistan in Norway. Over 41,000 inhabitants in Norway have Pakistani origin (immigrants from Pakistan and their descendants), which accounts for 3.8% of the population with a foreign background in Norway [14]. Immigrants from Pakistan are shown to have low participation rate in breast, cervical and colorectal cancer screening, and are thus potentially at risk for unfavorable outcome due to more advanced disease [8–10]. It is thus important to explore what immigrants from Pakistan know about CRC and how they perceive CRC screening. The aim of this study with qualitative interviews was to identify factors of importance for Pakistani immigrants’ participation in CRC screening.

## Theoretical framework

Health literacy is defined by the World Health Organization as what “represents the personal knowledge and competencies that accumulate through daily activities, social interactions and across generations. Personal knowledge and competencies are mediated by the organizational structures and availability of resources that enable people to access, understand, appraise and use information and services in ways that promote and maintain good health and well-being for themselves and those around them” [15].

Health literacy may be described as having literacy skills (such as reading and writing) and the capability to perform knowledge-based literacy tasks (such as acquiring, comprehending and applying health information) required to make health-related decisions in different environments [16]. It can also be categorized as functional, interactive and critical, describing what those literacies enable us to do [17].

Limited health literacy is associated with lower participation in health-promoting and disease detecting activities, riskier health choices and poorer health outcomes. Strengthening health literacy can contribute to reduce health inequities and enhance overall well-being [18]. Migrants tend to score lower on measures of literacy and health literacy, requiring other strategies to be reached [18, 19].

In this study we used health literacy as a framework to understand Pakistani immigrants’ thoughts and perspectives on CRC screening.

## Materials and methods

### Pakistani immigrants in Norway

The first group of Pakistani immigrants in Norway were young men who arrived in the late 60’s and early 70’s as labor migrants. Because of restrictions on immigration from the middle of the 70’s, those who arrived later primarily came through family immigration, as family members of the labor migrants [20]. More than half of the Pakistani immigrants arrived more than 20 years ago [21]. As a result, there are almost as many descendants of Pakistani immigrants as people who have immigrated themselves, and descendants of immigrants from Pakistan are the largest group among Norwegian-born people with immigrant parents [22].

The vast majority of Pakistani immigrants are Muslims, while three quarters of the Norwegian population are members of the Church of Norway [23]. Most Pakistani immigrants live in, or in close vicinity to, the capital city of Oslo. 37% of the women in working age are employed. This number is almost 70% among the female descendants of immigrants from Pakistan, being just slightly below the percentage for male descendants [22]. For comparison, 67% of women and 73% of men between 15 and 74 years in the total Norwegian population are employed [24]. Similar differences between the immigrants and their descendants can be seen in education. While the Pakistani immigrants have low educational status overall, their descendants have achieved higher education with a high proportion in professions such as medicine and law [18]. At the same time descendants of Pakistani immigrants have a higher dropout rate in high school compared to children of non-immigrant parents [18]. The Pakistani community is also represented in the public discourse in many fields, such as politics with politicians in government, law, health care, media, and culture.

While immigrants from Pakistan arrived as labor immigrants and did not plan to settle in Norway [20], Pakistani immigrants as a group have a long history in the country. However, they might not have been able to achieve higher education or a better knowledge of the Norwegian language and culture due to other priorities, such as economically supporting family and relatives in two countries and raising children in a foreign country. With that starting point and the fact that health-related information may not be equitably accessible to immigrants, it can be difficult to obtain a high level of health literacy [19]. If an organization fails to make the information equitably available for all parts of society, it will cause low organizational health literacy, which can lead to low personal health literacy. One must bear in mind that CRC screening has only recently been introduced in Norway and is not as well known in the general population as for instance mammographic screening.

## Study setting and participants

This paper is based on qualitative interviews with Pakistani immigrants in Norway (i.e. people who have immigrated to Norway themselves, and not children of immigrants) in the period of January to June 2023. There were twelve participants in total, six men and six women, all born in Pakistan (Table 1). Four of the participants immigrated as children and had most of their upbringing and schooling in Norway. The rest of them immigrated as young adults.

The participants were recruited by the interviewer, NI, who reached out to Pakistani associations in Oslo, connected with individuals through social media, and utilized her personal network. It is important to note that she did not have prior personal acquaintance with the participants. All participants lived in Oslo or within 1,5 hours’ driving distance from Oslo. One of the participants alternated between living in Norway and Pakistan. We attempted to include people living outside the southeastern part of Norway through the authors’ contact network but failed to reach them. It is worth mentioning that two-thirds of the Pakistani community in Norway live in Oslo [25]. We set the age group for inclusion of participants to 50–65 years while the target group for CRC screening program is 55–65 years. In this way, we included perspectives from those who were in the target age group for CRC screening as well as those who were due to reach the target group in a few years. Although the target group for CRC screening is every individual aged 55–65, the capacity for colonoscopies (for those with a positive test) is not sufficient to accommodate all individuals in this age range simultaneously. Therefore, the screening program began by including everyone who turned 55 in 2022 and adds one age cohort (those turning 55) each year. That’s why very few people in the target age group have participated in CRC screening (i.e. someone who was 58 years at the start of the screening program would not be invited). We succeeded in our attempt to get a diverse group in terms of gender, age, education, work, residence time in Norway and familiarity with the Norwegian language and culture (Table 1).

**Table 1:** Overview of the participants

		Total (*n* = 12)
**Gender**	Women	6
	Men	6
**Age distribution**	50–55	5
	56–60	2
	61–65	5
**Education**	Primary	5
	Secondary	1
	University	6
**Residence time in Norway (years)**	20-30	1
	31-40	2
	41–50	9
**Ever worked in Norway**	Yes	9
	No	3

### Qualitative interviews

All interviews were conducted by NI. She delivered the invitation letter and information material about CRC screening to the participants in person prior to the interviews. Two of a total of twelve interviews were conducted by phone, and these participants received the material by email. One of the participants interviewed by phone was in Pakistan at the time of the interview, and the other was residing far away. A third interview was initially conducted in person, but because we did not manage to get through all the topics and questions, we continued by phone the next day. The rest of the interviews were conducted in-person in a mosque, a library, at the Cancer Registry of Norway or premises that the local district had at its disposal. The participant and the interviewer were the only people present in the room during the interview. The interviews lasted for 40–150 min.

The interviews were conducted in Norwegian, Urdu or Punjabi. Both Urdu and Punjabi are languages commonly spoken in Pakistan. In addition, people from Pakistan regularly use English words when speaking Urdu or Punjabi. The participant chose the language he/she was most comfortable with. We had a semi-structured interview guide (supplementary 1) but did not follow it strictly as we wanted participants to follow their own thoughts and speak without unnecessary interruptions from the interviewer. It was communicated to the participants that we wanted the interview to be like a conversation and that we aspired to cover all the topics in the interview guide including background details, cancer, CRC, CRC screening and the health care system.

### First author

The first author, NI, is a Muslim female in her 30’s and a medical doctor by profession, doing her Ph.D. in cancer screening research. She is a descendant of Pakistani immigrants, born and raised in Norway, and considers herself being a part of the Norwegian-Pakistani community. NI speaks Norwegian, Urdu, and Punjabi fluently. We believe that she was perceived as one of “their own” or as an “insider” by the participants [26], which might have facilitated a more open exchange of thoughts. Being from the same culture, but a different generation, can contribute to gaining the emic perspective of the participants, as well as the etic perspective of the observer [27]. Being a medical doctor specializing in oncology might also have caused her to be perceived as an expert. The latter can facilitate asking questions or discussing things more comfortably, but can also lead to participants asking questions about their own health, striving to provide answers that they believe the interviewer wants to hear and cause hesitation in presenting less favorable health choices [28]. For example, when asked about participation in CRC screening, some participants responded by stating that they would participate because of health benefits. This can be interpreted as a correct response from a health care professional’s standpoint. Upon closer examination, it turned out that they were not inclined towards participation for various reasons. Nevertheless, it is important to remember that they may not have formed a clear opinion immediately upon receiving the question.

### Data analysis

All interviews were de-identified during transcription. NI listened to the interviews several times and translated the interviews sentence by sentence to English. Three of the interviews were conducted in Norwegian and transcribed by the software Autotekst and then edited by NI. SB who understands Urdu language listened and read through some of the interviews. NI was the main investigator and led the process of analyses. She discussed the findings with SB and PB and received written critical feedback from all authors.

We performed thematic analyses inspired by Braun and Clarke [25]. The transcripts were read through several times for familiarization. The data was then categorized in many unique codes using NVivo, a qualitative data analysis software. The codes are semantic focusing on the expressed meaning [29]. Through thematic analysis of the content, codes were consolidated into broader themes that were identified. Memos on codes and themes were written along the way to develop the final themes. Already during the interviews, it became apparent that many of the topics raised by the participants were somehow linked to health literacy. Furthermore, the theoretical framework of health literacy guided our work throughout the analysis; from reading the transcripts, through coding, to critical revision of the manuscript.

#### Ethical considerations

The Norwegian Data Protection Authority at Oslo University Hospital approved this study (20/15902). Informed consent was obtained from all participants. They were also informed about the possibility of having their information deleted at any time. We gave pseudonyms to all participants in the transcripts and in further analyses and presentation of the results. Audio recordings were deleted once the interviews were transcribed.

## Results

**Fig. 1:**
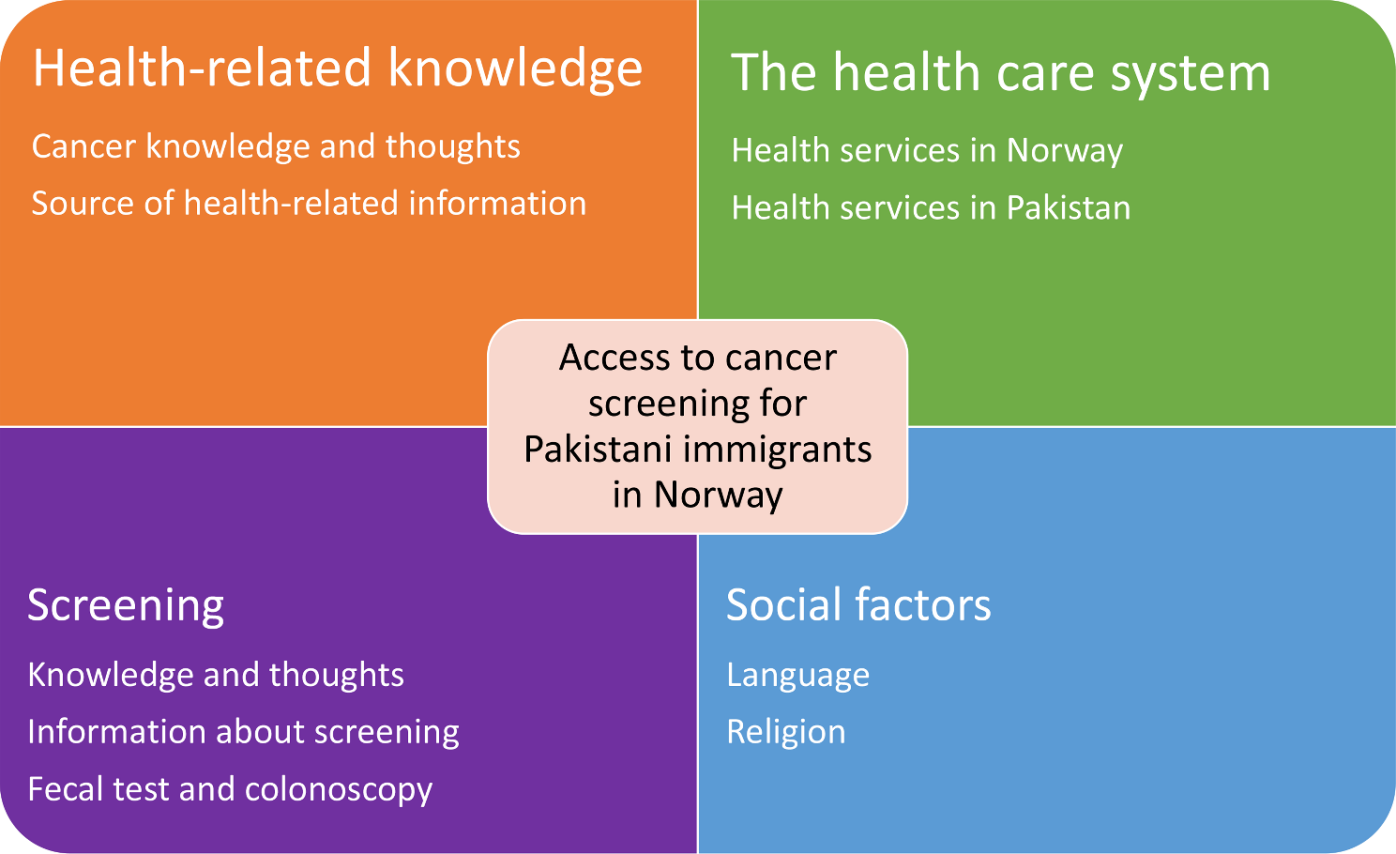
Themes and subthemes

We categorized our findings about the factors that influence Pakistani immigrants’ access to and attitudes towards CRC screening into four main themes that included up to three subthemes (Fig. 1). The main themes were health-related knowledge, the health care system, screening, and social factors.

### Health-related knowledge

#### Cancer knowledge and thoughts – cancer as “the unknown”

Cancer in general was perceived by some as terrifying and incurable, although most of the participants considered it treatable and curable. It was commonly heard of, and everyone had heard of a friend, relative or a family member with a history of cancer. Afzal described it like this:

It’s scary. I hear so often about friends and family and colleagues who have been affected by cancer unfortunately. Now it’s almost like heart disease that every third person has some type of cancer.

The majority had some knowledge about cancer in general even though they could not elaborate on how the disease develops. They had very little knowledge of CRC. Some could only relate it to a stoma. Many had thoughts about the causes. Razia suggested the diet and preparation of food being a cause:

It must be caused by what we eat. I think everything starts with that. Food that is not good and not prepared the right way. My whole life I have never heated milk for the children in the microwave. I have also made all the food at home.

One of the participants had read and knew a lot about the risk factors for CRC, precursor lesions, and polyps. Overall, the men volunteered more knowledge about prostate cancer and the women about breast cancer in comparison to CRC.

#### Source of health-related information – who to ask?

Participants who immigrated to Norway as children had the highest level of education. They could easily look up health-related information on the internet. Some of those who immigrated as adults could also easily look up information on the internet but most of them lacked digital literacy. Surraya elaborated:

People can google it. We can’t google and stuff like that. Now everything comes digitally which I often don’t see because it’s difficult for me.

The participants were dependent on asking someone and many suggested to ask their general practitioner (GP), but also children and friends with health-related professional backgrounds both in Norway and elsewhere were suggested as sources of information. Shehzad said:

Obviously I ask my GP. My wife or friends can’t give me health-related information. Yes, I consult my friends who are doctors or pharmacists.

Talking about where to get health-related information, Aisha explained how challenging it could be to seek written information:

That is the problem. We can’t get it from anywhere. Either I would ask a doctor I know in Pakistan or here. Or I would ask my own GP.

While Aisha suggested specific sources for health-related information, her knowledge about the variety of sources limited her possibility to obtain information.

### The health care system

#### Health services in Norway – navigating the system

Participation in CRC screening may lead to a closer interaction with the health care system in Norway. The participants’ view of the health care system is therefore of interest. All participants agreed on having a very good impression of the Norwegian health care system. The doctors were perceived as knowledgeable, professional, and trustworthy. Several of the participants compared the Norwegian and Pakistani health care system and expressed gratitude for being in the Norwegian system. Mushtaq said:

Compared to my own country it’s like being in Paradise. We get everything free. We just pay the deductible. In Pakistan a single blood test for uric acid costs 5000 rupees [€165].

It was mentioned that immigrants have limited access to information and are not aware of their rights. It was said that the lack of access to information made them passive in terms of health-related issues. Iqra explained:

Norwegians know of these things. But we don’t know what our rights are. And they [health care system/authorities] don’t tell us that much. If they tell us… like if we know then they will help us. I am not saying that they don’t help.

In Norway, the public health care system enables patients to access selected private specialists at the government’s expense, particularly when faced with prolonged waiting times within the public healt hcare system for specialist appointments [30]. Some participants criticized the long waiting lists in the public health care system. Those who had used private health services, usually through the public health care system due to long waiting lists, described the service as better. One participant said that the public health care system was overloaded and because of this they had a wait-and-see attitude which sometimes could be harmful for the patients who got help very late and ended up with long sick leave. Nazir said:

There are long waiting lists everywhere no matter where you go. It takes a bit too long for someone to get admitted to the hospital to get some examinations. But the challenge is suddenly that you get so bad that it takes longer to get well too. If you had been there early it would have been much easier.

Some of the participants mentioned that the doctors have very little time for each patient and each problem. The participants also found it challenging to get the GP to take a holistic approach and mentioned that the GP hardly told the patients more than they asked.

#### Health services in Pakistan – navigating in chaos

Everyone had different experiences regarding health services in Pakistan but very few had sought health services unless they needed doing so because of acute sickness. Usually, it was preferred to get back to Norway in case of sickness. As Zaid said:

We try to avoid seeking help there. My closest relatives, when they have occasionally had health problems in Pakistan, they want to get to Norway as soon as possible to get help. That’s what we do.

The health care system in Pakistan was not considered trustworthy. Many pointed out money as the incentive when treatment, such as surgery, was recommended. Others spoke well of the private health market but emphasized that you must pay for it. The doctors in Pakistan were perceived as very knowledgeable and with a lot of experience. Some had experienced getting optimal treatment for diseases in Pakistan which doctors in Norway were not able to diagnose, but the system was inadequate. Asia explained:

I had an eye disease which I don’t know where I got from. I consulted doctors in Norway. No one understood what it was. My eye was red and painful all the time. Then I consulted a doctor in Pakistan and he said it will take six months and you will be free from this virus. Nothing helps, no medicine, no drops. And you know, on the date after six months that virus was over and out.

One of the participants had used public health services in Pakistan. His experience was that you meet very knowledgeable doctors, the same doctors and professors who ran expensive private clinics after work hours. But he explained that you must be in unhygienic surroundings and were dependent on next of kin to help you. Bashir said:

The doctors in Pakistan have tried and failed much more than what they do in Norway. The health care personnel in Norway are extremely careful. They will safeguard themselves from everything. And you can’t learn when you are safeguarding yourself from everything. You learn when it’s chaos and you just must do something.

As he saw it, Pakistani doctors were less concerned about safeguarding themselves compared to Norwegian doctors, which gave Pakistani doctors better possibilities to gain experience. Doctors in Pakistan worked in a much more hectic environment where they are thrown into unfamiliar challenges they must solve. This, he elaborated, resulted in doctors in Pakistan lacking the time to fully engage with patients, instead they viewed each patient as a case.

### Screening

#### Knowledge and thoughts – considering participation

The participants spoke positively about screening on a general basis. But the overall impression was that several of the participants were finding it difficult to understand what screening really is. Some seemed to confuse screening with treatment. Like Zainab:

We should participate in screening. If there is any other precaution, we should take that as well. I am for treatment in any form.

Most of the participants agreed on screening being beneficial. They explained that it can cause early detection of cancer and one can avoid metastases, that cancer is easier to treat in an early stage and enables you to live longer. Some participants emphasized the importance of ruling out cancer. But there was also fear related to screening. Like Noor expressed:

You feel like it’s judgment day, let’s say every other year. That you are put on a very difficult test, just waiting for the result. And then you have to go through that again every other year.

There were other barriers as well following a positive FIT, such as the gender of the examinator when it came to colonoscopy and (partial) nudity. Both men and women experienced discomfort in that regard. The hesitation regarding gender was mainly regarding screening examinations. It was less of an issue when they were getting treatment for a disease which was already diagnosed.

#### Information about screening – mind the communication method

Some of the participants could easily read and understand the invitation letter and information material in Norwegian about CRC screening. They found it useful and balanced in terms of the amount of information. One of them asked for even more information and details. Others had difficulties with reading and understanding the written material. They needed help to understand the information. Some asked their friends who were health care professionals, but the majority asked their children to help. The help they received varied. As Shabana explained:

When you talk to the children they say; it’s nothing special. Leave it they say. Whenever something like that comes and it’s difficult and I must get the children to read it. They say leave it, it’s nothing special.

But some had children who were health care professionals and encouraged their parents to participate. It seemed like less of an issue that the letter was in Norwegian, even though some of the participants said they would have been able to read it better if it was in Urdu. The letter was also perceived to be too long. Kareem suggested keeping it short and concise:

They should keep it short. One-page, clear cut. Like if these are the symptoms, do this.

A few also admitted that they neither would have read the letter themselves nor would they have asked for help. Oral information was suggested as an alternative, which most of the participants were positive about. Some suggested informing at gatherings in the language of the participants. That way it would be possible to ask questions as well. The participants said that it would be an advantage to have heard about CRC screening prior to the letter, which could increase the probability of participation. Salma elaborated:

In our age oral information will have more impact than written. Many of us can’t even read Norwegian. Orally and in your own language is good, like it’s good to talk to you. I can ask anything I want and I am getting answers. When I get the letter, I will remember that I have heard something about this.

Most of the participants believed the GP should be involved in some way. Some pointed out that the GP had authority which was important to get people to participate. It was preferred that the GP should be the one to arrange an appointment where participants could get information and take the test. Alternatively, it was suggested that the GP at least gave information about CRC screening, or that the participants could take the test at the GP’s office after receiving the letter. That way, the GP would have some kind of involvement in CRC screening and would be able to guide the participants. Saddiq said:

To be honest, the GP is the one with the most important role. If your GP informs you of something, that would be good, not only for me but for his other patients as well. It’s good for everyone. Because the doctor is a big motivator. For everyone.

The participants put a lot of trust in the GP. The majority had an Urdu-speaking GP so they could communicate easily. There are many Urdu-speaking GPs in the Oslo-area which makes it convenient to have a GP speaking the same language and having the same cultural background.

#### Fecal test and colonoscopy – when is it necessary?

The fecal test seemed to be too laborious for many of the participants. Even those who had grown up in Norway and understood Norwegian language well found it complicated, with many steps, and hard to complete. The overall impression of the interviewer was that many participants had not read the instruction manual and were answering the questions hypothetically. Like Kiran:

I don’t understand everything but I guess when you start the procedure you will understand what to do next by yourself. I would do it, must do it, for my own health. But I don’t know if I would actually do it or not right now.

Others were very clear that they would not have participated. One of them said that we receive many letters and hear of many tests. But he did not feel like spending time on it without any symptoms. He said:

If you have symptoms, then you will take it seriously. If not, then you will say why would I get in all this trouble. I would think it’s complicated and dirty. Leave it. Nothing is wrong with me.

Many expressed that it would be easier to participate after our conversation. They appreciated all the information about the test and answers to their questions.

A few of the participants knew about colonoscopy. A couple of them had had a colonoscopy as a diagnostic test. Several of them had had gastroscopy which they remembered as an uncomfortable examination. All participants stated that they would have done a colonoscopy after a positive fecal test. They considered the examination necessary at that point. However, the threshold was higher to participate in CRC screening if it had been through a colonoscopy without blood detected in feces prior to the examination. Only a couple said that they would have participated in such a scenario, as explained by Nusrat:

When blood is detected then you must go through the process. There is no other way out. But if it was not required and I had no symptoms, then I would have cancelled the appointment.

One of the participants asked for more information about potential complications during and after a colonoscopy and how they could be treated.

### Social factors

#### Language – the basis of all communication

The information and invitation letters of the CRC screening program are written in Norwegian. Most of the participants understood Norwegian well. One of the participants said she could not speak Norwegian at all despite living in Norway for many years. Most of the participants still needed help to read and understand health-related information. Nimra explained:

When I get a letter, whenever the children have time, they read it to me. I can’t understand it myself. Especially the special letters. Not everyone can understand their language or what it is.

On the other hand, those who immigrated to Norway as children understood Norwegian very well, but they did not understand Urdu in the same way. Written Urdu was especially challenging as many of them could not read Urdu at all. Naveed, who grew up in Norway, said:

We had an Urdu teacher on the weekends, but we just fooled around and then went home. So I can’t read Urdu, but oral communication is not a problem.

Pakistani immigrants constitute a diverse group with varying levels of understanding of their mother tongue. These results point out that while it may be easier for some Pakistani immigrants to receive information in their mother tongue, that information would be entirely incomprehensible to others who cannot read their mother tongue.

#### Religion – who makes the choice, god or me?

All participants were Muslims, but they practiced religion to varying degrees. Some participants were of the conviction that the day they will die was predetermined. It was not possible to extend life in any way. So, if it is predetermined that someone will die of cancer then you cannot prevent it by screening. Hiba explained:

I believe that the day God has decided that I will die, that day I will die. No one can take that away from me. I know that no one can give me one second more or less.

But at the same time, those who had that conviction also emphasized that their religion instructs them to take care of their health. Although they differed on whether screening is a part of taking care of one’s health or not. It was also mentioned that human beings have free will and make choices all the time. Participation in cancer screening was also considered a choice. Hakeem said:

Things depend on our choices. Because ultimately what have you done to prevent it? If you are taking care of your health by taking a test every other year, then that is a part of your religion.

Religion was usually not a hindrance to screening, and for some, it was even an encouragement. However, one had to reflect a bit to reach that conclusion.

## Discussion

In this qualitative study based on interviews with twelve immigrants from Pakistan living in Norway, we identified several factors influencing Pakistani immigrants’ participation in CRC screening. We used health literacy as the theoretical framework and presented our results in four themes of relevance to perspectives and participation in CRC screening: health-related knowledge, the health care system, screening, and social factors. This is the first study exploring factors of importance for Pakistani immigrants’ participation in CRC screening in Norway and one of few studies exploring factors of importance for immigrants’ participation in CRC screening in Europe.

Studies conducted in UK exploring barriers to CRC screening among immigrants, including Pakistani immigrants, reported limited awareness, lack of symptoms and cultural factors [31, 32]. A study from the Netherlands exploring knowledge, attitudes and beliefs regarding CRC and CRC screening among ethnic minority groups concluded that language barrier and low literacy were serious barriers to informed participation in CRC screening [33]. Observations in these studies align with our findings. There is one Norwegian study exploring Polish immigrants’ access to CRC screening. There are many differences between the Polish and Pakistani immigrants in Norway, including the practice of traveling to the country of origin for a colonoscopy [34]. Nevertheless, the significance of the GP, and use of non-public sources for information, were observed in both groups.

As described in previous studies, knowledge is a key element for screening attendance. Poor knowledge of CRC and CRC screening is associated with lower CRC screening rates among South Asian immigrants in North America and England [35–38]. Basic cancer attributes were often easily understood by participants in our study, such as cancer being a potentially lethal but also potentially curable disease. As knowledge advances, its complexity increases, demanding a higher level of comprehension and necessitating a higher level of health literacy. High level of health literacy is dependent on overall literacy, underscoring education as a pivotal factor [18]. However, even people with a high level of education can be at risk of misconceptions about health information, especially when the topic is complex [39]. Organizational health literacy is therefore crucial. If information is not made comprehensible and readily accessible to everyone, only those possessing the necessary skills will actively seek it. For instance, online information may be accessible only to individuals with digital literacy. An earlier qualitative study showed that immigrants from Pakistan had difficulties accessing digital information in Urdu on BreastScreen Norway’s website [40]. Accessing eHealth services may pose challenges for those lacking digital skills. Research indicates a disparity within the population regarding the ability to utilize eHealth services [41]. A high level of eHealth literacy might be even more demanding than health literacy, as eHealth literacy includes both health literacy as well as five other types of literacy [42].

Accessibility can be conceptualized with five dimensions; approachability, acceptability, availability and accommodation, affordability and appropriateness [43]. Achieving accessibility depends on both personal and organizational health literacy. In our study we found that individuals with limited education and lack of digital competence were more dependent on asking someone in their network to access information. They were aware of their inability to access information on their own. One participant suggested that limited digital literacy was the reason for their passivity regarding health-related issues. Another asked for a straight-forward recommendation. The demand for simplified information has been reported earlier [40].

The health care system emerged as a theme. Preventive health measures such as CRC screening can be perceived as part of the health care system. The differences between health services in Norway and Pakistan are numerous. A well-established universal public health care system in Norway, which is not driven by financial motives, was preferred by the participants in our study. However, there was a need for guidance in navigating the system and a request for a more holistic approach. In accordance with other studies, the GP was singled out as a particularly important facilitator [36, 44]. There could be many reasons for wanting active involvement from the GP. Many participants had GPs with the same cultural background as themselves with whom they could communicate in their mother tongue. Moreover, the communication with the GP was conducted orally. Individuals from countries with a different mother tongue than the majority population often tend to favor verbal or visual channels, as well as face-to-face communication [45]. A Norwegian study investigated whether an intervention among GPs could influence immigrant women’s participation in cervical cancer screening program and found a significant increase in cervical cancer screening participation among immigrants [46]. The intervention particularly increased participation for some groups including women from Pakistan.

A study from the USA showed that behavior preventing diseases was not prioritized by South Asian Muslims [36]. CRC is a disease that often presents symptoms in late stage, and secondary prevention, such as screening, is therefore cruical for CRC. Our findings support that preventive health behavior has low priority among Pakistani immigrants in Norway. There was little knowledge and awareness about the concept of screening and the goal of CRC screening. This is similar to what has been found in other studies with immigrants [33, 44, 47]. The absence of symptoms led to the perception that screening was redundant. If one does not understand the purpose of screening and relies on assistance to comprehend and complete the test, the threshold for participation becomes high. All participants had a positive attitude towards colonoscopy when the risk of illness was concrete, for instance when blood is already detected in the stool, even though it is a more invasive procedure. However, a recent study showed that non-Western immigrants had lower participation than non-immigrants in colonoscopy after a positive FIT [10].

Our findings suggest that social factors, such as language and religion, are of relevance for participation in CRC screening to some extent, but these factors alone seem unlikely to explain non-participation in screening. However, the findings in our study show that they are intertwined and involved in a complex interplay during a decision-making process. If the basic understanding of screening is not present, participation can be challenging, and social factors may be of influence. Language, for example, is fundamental in all communication. However, with information about CRC screening in Norwegian or translated into your mother tongue, you may still lean towards non-participation due to a lack of understanding of the purpose. In such a scenario, the focus might be on the symptoms or the absence thereof, weakening the perceived necessity for a test or examination.

Cancer screening programs strive to be equally accessible to all segments of society. The service providers aim to achieve equal access with participation rates higher than a pre-specified level across diverse societal groups. The primary objective, however, is to empower every individual, including immigrants, with the opportunity to make informed decisions. The ultimate goal should thus be *equitable* access to cancer screening programs. Screening organizations in many countries, including Norway, are trying to improve access for immigrants by translating written and video materials into multiple languages, as well as conducting quantitative and qualitative research on participation rates among immigrants and other at-risk groups [48]. However, BreastScreen Norway have performed an RCT in which immigrants had the same participation whether they received invitations for screening in Norwegian only or Norwegian and their mother tongue [49].

The question is though, how to decide whether informed decision making is achieved regardless of participation. In our study we found that adult children often make the decisions for their parents. Hence, the parents have not autonomously made an informed decision regarding their own participation. Instead, they rely on the viewpoints and health literacy of others. Oral information in mother tongue was suggested by many participants, both in our study and others [33]. In 2009–2010 several public health offices and non-governmental organizations collaborated in campaigns with outreach activities interacting with the Norwegian-Pakistani community, informing Pakistani immigrants about breast cancer and breast cancer screening. The campaigns made little impact on mammographic screening attendance for Pakistani immigrants in Norway (participation rate increased from 32 to 36%) [8]. However, the campaigns were limited in terms of both frequency and the number of locations where such activities took place [50]. This raises the question whether oral information in mother tongue increased the women’s possibility to make an informed decision about participation in breast cancer screening. Our final point is the recognition of the possibility that individuals, through informed decisions, may choose not to participate. Regardless, service providers must strive to enable everyone to make an informed choice.

### Limitations

While we included a diverse group of participants in our study, there are several factors that may challenge the trustworthiness of our findings. All our participants were residing in the Southeast part of Norway. While there are smaller communities of people with Pakistani family background in other parts of Norway that might have other experiences, most of the Pakistani community in Norway resides in the greater Oslo area. Therefore, our participants with varied sociodemographic backgrounds can represent the majority of this population.

Our participants, along with those in a previous study on mammographic screening among Pakistani immigrants, have described the important role that children of immigrants play in their decisions regarding health-related issues [40]. One could thus argue that we should have interviewed some children of immigrants as well as the immigrants themselves. An insight into their thoughts and perceptions could be of importance. Nevertheless, we believe to have brought forth many nuances by giving the participants the opportunity to communicate in their mother tongue. Further, some of our participants immigrated to Norway as children, and may be considered both as immigrants and children of immigrants.

Our study focuses on Pakistani immigrants in Norway and our findings cannot uncritically be transferred to other settings. However, we believe that some of our findings may be transferrable to other immigrant groups in Norway and Pakistani immigrants in other countries, and that service providers working with such groups can make use of the knowledge gained in our study. Through a rigorous process that started with a detailed protocol before the interviews and involved all the co-authors throughout a thorough process of analysis, writing up the manuscript and critical revision, we believe that we present findings that are grounded in the data. Two of our participants have also reviewed our findings as member checking.

## Conclusion

There are many factors of importance regarding Pakistani immigrants’ participation in CRC screening. Recommendations from the GP and the participants’ close social circle including adult children are particularly important. We consider knowledge and basic understanding of the CRC screening program to be essential for participation. Increasing health literacy among immigrants might increase the ability to make informed choices. This could be the primary objective of future interventions. Our study should be of interest to policy makers, service providers and health professionals communicating with immigrants and those who are working with topics related to migrant health.

### Electronic supplementary material

Below is the link to the electronic supplementary material.

Supplementary Material 1

## Data Availability

Data sharing is not applicable to this article as no quantitative datasets were generated or analyzed during the current study. Qualitative data generated from this study are included in this published article.

## Abbreviations

CRC: Colorectal Cancer
FIT: Fecal immunochemical test
GP: General practitioner
