# Supplementary material for: Access to colorectal cancer screening for Pakistani immigrants in Norway – a qualitative study
Source: BMC Health Serv Res. 2024 Jul 11;24:799. doi: 10.1186/s12913-024-11275-7 (PMC11238370; doi:10.1186/s12913-024-11275-7)
Supplement: Supplementary file 1 — Supplementary Material 1 [file 12913_2024_11275_MOESM1_ESM.docx]

Supplementary material

Supplementary 1: Interview Guide

**Before the interview**

Introduction about yourself/your team and some information about the project: We are interested in understanding what Pakistanis think about colorectal cancer (CRC) and CRC screening. Which factors influence their decision to participate or not.

Emphasize that research data will be used anonymously.

Send invitation- and information letters in advance so the participant has had time to review them. Must be done personally or by mail for those who do not use email.

**During the interview**

Begin by providing some information about yourself/your team and the project.

Obtain consent for participation in the interview and recording.

Background

- Name, age, spouse/children (or other family members the participant lives with - parents, grandchildren).
- Country of birth (city or village), length of stay in Norway, reason for emigration.
- Education, current job/previous jobs (in Norway or abroad).
- Your role in the household/typical household chores. Leisure activities.
- How often do you visit Pakistan and is it for vacation or other obligations?
- Do you use healthcare services in Pakistan? How does it compare to using healthcare services in Norway?

Cancer and CRC

- What comes to your mind when I mention cancer?
- Do you know anyone who has or has had cancer? If yes, what type?
- What do you know about CRC?
- What do you think can be done to avoid getting cancer or to detect it at an early stage?
- What are the benefits of detecting cancer early? What are the disadvantages?

CRC screening

Provide some information about the CRC screening program.

Present the colorectal screening invitation.

- What do you think about such an invitation?
- Would you have read it if it came in the mail? Why/why not?
- Do you understand the information? Is there anything missing?
- Would you participate if such an invitation came in the mail?
- Do you understand how to carry out the fecal immunochemical test?
- Do you know what a colonoscopy is and what do you think about such an examination?

Information/health care system

- Do you find it satisfactory to receive information this way?
- How would you prefer to receive such information?
- What do you think about the role of the general practitioner regarding CRC screening?
- What do you think about the Norwegian healthcare system (and Norwegian doctors)?
- Where do you naturally seek health-related information?

Conclusion

- Do you have any questions/comments?
- Would you be interested in participating in a follow-up interview?
- Contact information.

Thank you very much!
